# Chemical profiling of root bark extract from *Oplopanax elatus* and its *in vitro* biotransformation by human intestinal microbiota

**DOI:** 10.7717/peerj.12513

**Published:** 2021-11-24

**Authors:** Jin-Yi Wan, Jing-Xuan Wan, Shilei Wang, Xiaolu Wang, Wenqian Guo, Han Ma, Yuqi Wu, Chong-Zhi Wang, Lian-Wen Qi, Ping Li, Haiqiang Yao, Chun-Su Yuan

**Affiliations:** 1 School of Traditional Chinese Medicine & National Institute of TCM Constitution and Preventive Medicine, Beijing University of Chinese Medicine, Beijing, China; 2 State Key Laboratory of Natural Medicines, China Pharmaceutical University, Nanjing, China; 3 Tang Center for Herbal Medicine Research & Department of Anesthesia and Critical Care, University of Chicago, Chicago, IL, USA

**Keywords:** *Oplopanax elatus*, Intestinal microbiota, UPLC-Q-TOF/MS, Metabolic profiles, Biotransformation

## Abstract

*Oplopanax elatus* (Nakai) Nakai, in the Araliaceae family, has been used in traditional Chinese medicine (TCM) to treat diseases as an adaptogen for thousands of years. This study established an ultra-performance liquid chromatography coupled with quadrupole time-of-flight tandem mass spectrometry (UPLC-Q-TOF/MS) method to identify chemical components and biotransformation metabolites of root bark extract from *O. elatus*. A total of 18 compounds were characterized in *O. elatus* extract, and 62 metabolites by human intestinal microbiota were detected. Two polyynes, falcarindiol and oplopandiol were recognized as the main components of *O. elatus*, whose metabolites are further illustrated. Several metabolic pathways were proposed to generate the detected metabolites, including methylation, hydrogenation, demethylation, dehydroxylation, and hydroxylation. These findings indicated that intestinal microbiota might play an essential role in mediating the bioactivity of *O. elatus*.

## Introduction

*Oplopanax elatus* (Nakai) Nakai is the plant of genus *Oplopanax*, which belongs to the Araliaceae family. It is mainly distributed in northeast China, Korea and far east of Russia (Dou et al., 2009; Yang et al., 2010). As a traditional medicinal plant, *O. elatus* is being utilized as a ginseng-like herbal medicine and has been long used as an adaptogen to treat arthritis, diabetes mellitus, rheumatism, neurasthenia, and cardiovascular diseases (Dai et al., 2016; Eom et al., 2017; Knispel et al., 2013; Moon et al., 2013; Panossian et al., 2021). Previous studies have identified several components derived from *O. elatus*, such as the lignans, saponins, phenolic glycosides, and polyynes (Huang et al., 2010; Shao et al., 2016). To date, polyynes have been chiefly reported with high contents in the root of *O. elatus* (Huang et al., 2014a). Increasing attention has been paid to two main polyynes facarindiol (FAD) and oplopandiol (OPD), because of their significant anti-tumor activities (Purup, Larsen & Christensen, 2009; Qiao et al., 2017; Sun et al., 2016). However, most studies remain focused on the pharmacological and chemical constituents of *O. elatus*, while its metabolic profiles are rather obscured.

It is widely known that human beings live in symbiotics with coevolutionary microbiota (Thursby & Juge, 2017; Yang & Lao, 2019). The human gastrointestinal tract is the primary habitat for trillions of microbes. The gut microbiota serves metabolic functions crucial for the human host (Bäckhed et al., 2004; Chen et al., 2016; Rajilić-Stojanović & de Vos, 2014) and influences the biofunctions (Barko et al., 2018; Defois et al., 2018; Pagliari et al., 2017). Like most herbal medicines, *O. elatus* products are orally administered. The multiple constituents of *O. elatus* are typically brought into contact with intestinal bacteria and subsequently transformed in the digestive tract (Gao et al., 2018; Huang et al., 2014b; Koppel, Maini Rekdal & Balskus, 2017; Shikov et al., 2014; Teschke et al., 2015). However, existing reports did not address intestinal microflora’s biotransformed metabolites of *O. elatus*. Therefore, elucidating how gut microbes treat these complex components may contribute to a complete understanding of the metabolic profiles and biological activities of *O. elatus*.

Recently, various analytical platforms are typically applied to identify metabolic profiles in the complex extracts of TCMs. Most notably, ultra-performance liquid chromatography coupled with quadrupole time-of-flight mass spectrometry (UPLC-Q-TOF-MS) is one of the powerful analytical tools (Jin et al., 2018; Wu et al., 2019; Yang et al., 2016). With the newly developed chromatographic technique, the UPLC system allows significant improvements in the resolution, analysis speed, and reduction of solvent waste (Chekmeneva et al., 2018; Du et al., 2017; Wang et al., 2008). Meanwhile, high-resolution Q-TOF/MS can give more specific and accurate mass information on characteristic molecular ions and fragment ions, providing a reliable basis for the qualitative analysis of complex samples (Li et al., 2019; Lou et al., 2015; Wewer et al., 2011). Based on these characteristics, UPLC-Q-TOF/MS was ultimately selected for fast identification of constituents in *O. elatus*.

In the present study, we focused on the metabolic behavior of human intestinal microflora on *O. elatus*. A highly selective and sensitive UPLC-Q-TOF/MS method was established to characterize the chemical and metabolic profiles of *O. elatus*. Furthermore, the proposed metabolic pathways were also summarized. This work will provide a better understanding for exploring the bioactivities of *O. elatus in vivo*.

## Materials & methods

### Materials and reagents

The general anaerobic medium for bacteria culture was obtained from Shanghai Kayon Biological Technology Co. Ltd. (Shanghai, China). Formic acid and HPLC-grade acetonitrile were purchased from Merck (Darmstadt, Germany). Deionized water (18 MΩ·cm) was supplied with a Millipore Milli-Q water system (Milford, MA, USA). All other reagents were from standard commercial sources and of analytical purity.

### Preparation of *Oplopanax elatus* extract

Root bark of *O. elatus* was obtained from Benxi city (Liaoning, China). The voucher samples were deposited at the Tang Center for Herbal Medicine Research at the University of Chicago (Chicago, IL, USA). The air-dried root bark of *O. elatus* was pulverized into powder and sieved through an 80-mesh screen. Eight g of the powder were extracted twice by heat-reflux with 70% ethanol for 2 h. The combined extract was evaporated under vacuum and lyophilized with a yield of 28%. The samples were stored at 4 °C until use.

### Preparation of human intestinal microflora

The Institutional Review Board approved the present study protocol at the University of Chicago (IRB protocol number: 12536). Fresh fecal samples were collected from six healthy adult volunteers (male, aged 20–55, non-smokers without antibiotic consumption for more than 6 months, and written consent was obtained). All the fecal samples were mixed for analysis. A total of five g of samples were homogenized in 30 ml cold physiological saline, and centrifuged at 13,000 rpm for 10 min to obtain the resulting fecal supernatant.

### Incubation of sample in intestinal bacteria

Two microliters of the fecal supernatant were added with eight ml anaerobic dilution medium containing five mg of *O. elatus* extract, which were then anaerobically incubated at 37 °C for 24 h in an anaerobic workstation (Electrotek, UK). The reaction mixtures were extracted three times with water-saturated n-butanol. All the n-butanol layers were mixed and dried under a nitrogen stream and then dissolved in one ml methanol. The solutions were centrifuged at 13,000 rpm for 10 min for analysis.

### UPLC-Q-TOF/MS analysis

Data were collected as previously described (Wang et al., 2020). The Agilent 1290 Series UPLC system (Agilent Technologies, Santa Clara, CA, USA) was applied to perform the chromatographic analysis, and a binary pump, an online degasser, an auto plate-sampler, and a thermostatically controlled column compartment were also equipped for this system. The separation was carried out on UPLC ACQUITY HSS C_8_ column (2.1 mm × 100 mm × 1.7 μm, Waters) with a constant flow rate of 0.4 mL/min, and the column temperature was kept at 40 °C. A gradient mobile phase system of 0.1% formic acid in water (phase A) and acetonitrile (phase B) was applied as follows: 5% B at 0–1 min, 5–20% B at 1–18 min, 20–30% B at 18–27 min, 30–35% B at 27–32 min, 35–60% B at 32–40 min, 60–95% B at 40–50 min, 95% B at 50–53 min, 95–5% B at 53–55 min. The injection volume of samples was set at two μL for MS mode and five μL for MS/MS mode.

The Agilent 6545 Q-TOF-MS system with a Dual electrospray ionization source was used to conduct the detection. Nitrogen (purity > 99.999%) served as a sheath gas and drying gas, and the flow velocities were set at 11 and 8 L/min. The temperatures of sheath gas and drying gas were set at 350 and 320 °C respectively. Positive and negative ion modes were both employed in this study. The other parameters were set as follows: nebulizer pressure, 35 psig; voltage, 3,500 V; fragmentor voltage, 175 V; mass range, *m/z* 100–1,700; data acquisition rate, 1.5 scans/s; MS/MS spectra collision energy, 50 eV (Wang et al., 2020).

### Data analysis

Mass data were analyzed by the Agilent MassHunter Workstation software (Version B.06.01), based on the accurate measurements of *m/z* values with online databases (MassBank, etc.), to screen probable compounds. The empirical molecular formula was deduced by comparing the theoretical mass of molecular ions at the mass accuracy of less than five ppm.

## Results

### Optimization of UPLC-Q-TOF/MS conditions

To obtain the chromatograms with better resolution and higher baseline stability of *O. elatus* extract and its primary metabolites, multiple mobile phases such as acetonitrile-water and methanol-water were detected. Acetonitrile-water was applied as the solvent, for its stronger separation ability, shorter retention time, and lower column pressure. Additionally, 0.1% formic acid added in the water as mobile phase adducts may help to achieve higher response and better peak sensitivity (Tao et al., 2016). Therefore, the optimal solvent system consisting of acetonitrile-water (0.1% formic acid), which remarkably enhanced the efficiency of ionization and satisfactory sensitivity, was ultimately selected as mobile phase with a gradient elution.

In addition, the factors related to MS performance, including ionization mode and collision energy, were further improved. The positive ion mode was ultimately employed to gain comprehensive data for structural characterization and metabolite assignment with much lower background noise. The collision energy was optimized to obtain the higher ionization efficiency and relative abundance of precursor and product ions.

### Chemical profiling of *O. elatus* extract

**Figure 1:**
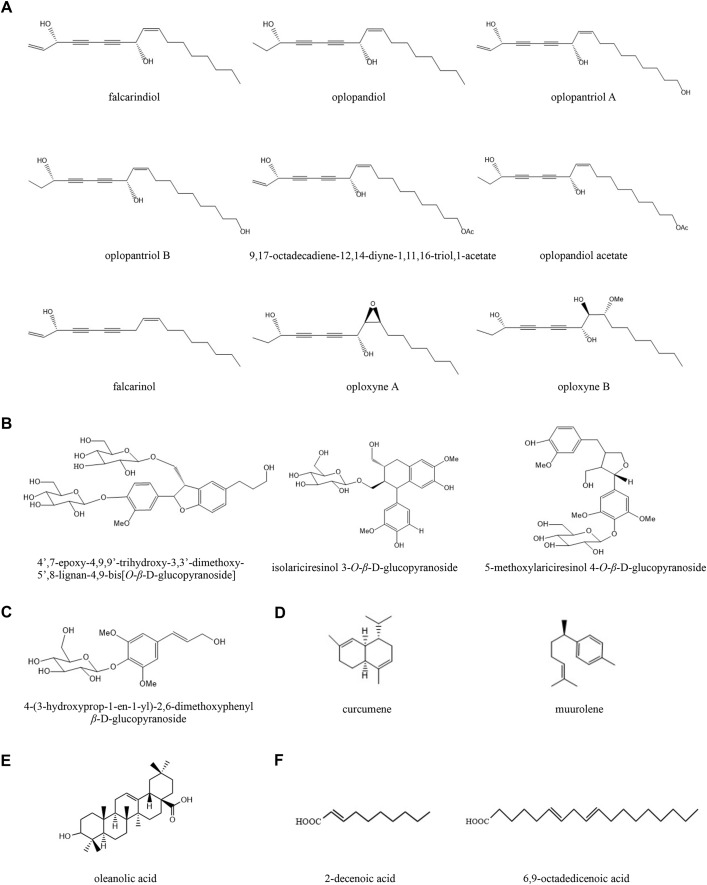
The chemical structures of bioactive compounds detected in *Oplopanax elatus* extract. (A) Polyynes; (B) Lignans; (C) Phenylpropanoid; (D) Sesquiterpenes; (E) Triterpenoid; (F) Fatty acids.

**Figure 2:**
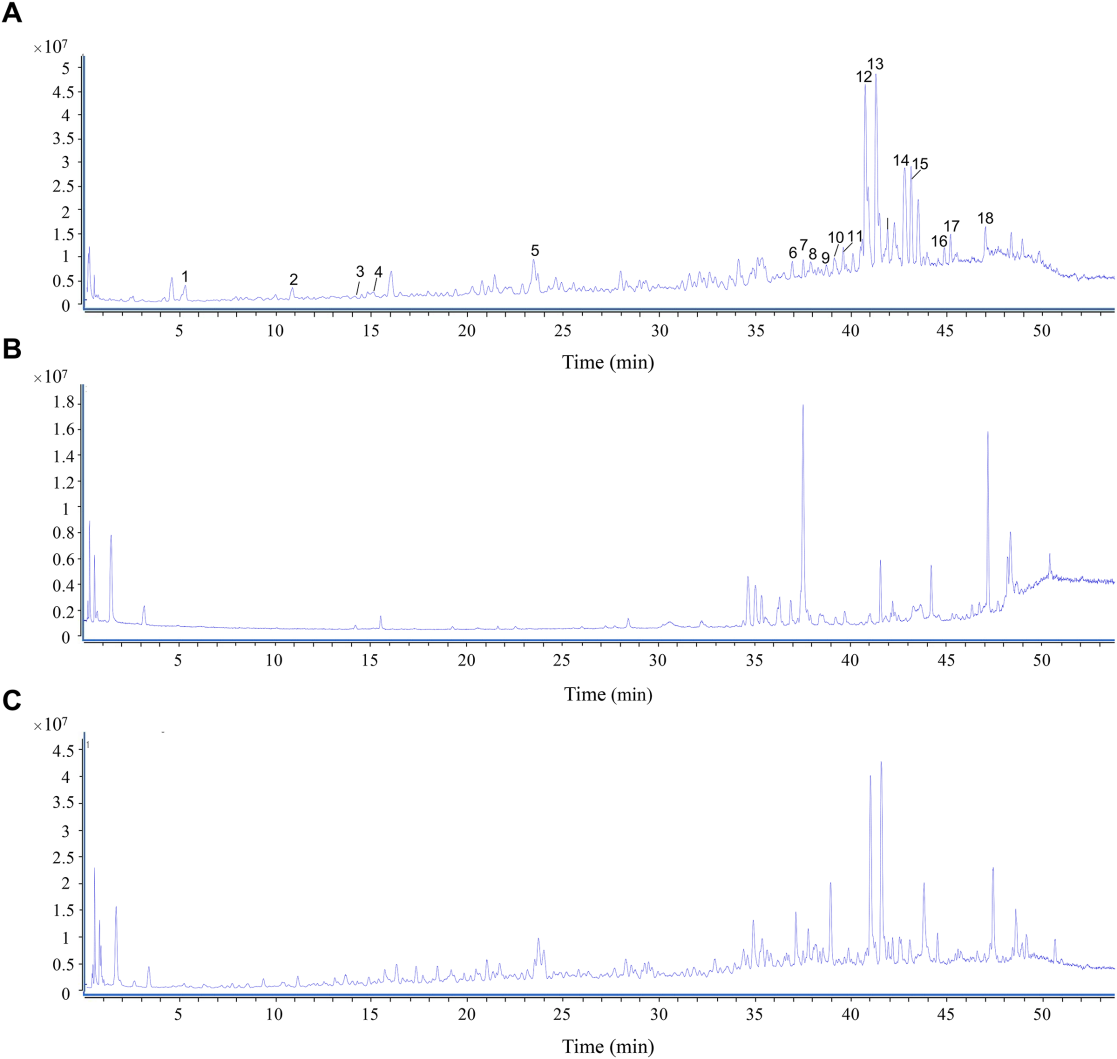
UPLC-TOF/MS profiles of *O. elatus* extract in the positive ion mode. (A) Total ion chromatogram (TIC) of *O. elatus* extract. (B) TIC of blank sample including dilution medium and human fecal microflora. (C) TIC of biotransformed *O. elatus* sample by intestinal bacteria.

In total, 18 ingredients of *O. elatus* were detected in this study, and their chemical structures are shown in Fig. 1. There are six types of compounds, including nine polyynes, three lignans, one phenylpropanoid, two sesquiterpenes, one triterpenoid, and two fatty acids. The total ion chromatogram (TIC) of *O. elatus* extract is shown in Fig. 2A in the positive ion mode by UPLC-Q-TOF-MS. Table 1 shows the detailed information, including retention time, signal intensity, molecular formula, calculated and experimental mass *m/z*, ppm error, and fragment ions of these 18 components (Schymanski et al., 2014; Wang et al., 2020).

**Table 1:** UPLC-Q-TOF/MS data of bioactive components of *O. elatus* extract in the positive ion mode (Dou et al., 2009; Shikov et al., 2014; Wang et al., 2020).

No.	Compound	Formula	t_R_ (min)	Signal intensity (×10^5^)	[M+H]^+^ or [M+Na]^+^			Fragment ions in the positive mode with the energy 50 V CID
					*m/z*	Calc *m/z*	Diff (ppm)	
1	4-(3-hydroxyprop-1-en-1-yl)-2,6-dimethoxyphenyl *β*-D-glucopyranoside	C_17_H_24_O_9_	5.24	2.86 ± 0.82	373.1495	373.1493	−0.51	211.1526[M-glc+H]^+^, 373.1495[M+H]^+^
2	4’,7-epoxy-4,9,9’-trihydroxy-3,3’-dimethoxy-5’,8-lignan-4,9-bis[*O*-*β*-D-glucopyranoside]	C_32_H_44_O_16_	10.92	1.74 ± 0.34	707.2521	707.2522	0.08	545.1981[M-glc+Na]^+^, 707.2521[M+Na]^+^
3	isolariciresinol 3-*O*-*β*-D-glucopyranoside	C_26_H_34_O_11_	14.14	0.55 ± 0.17	545.1997	545.1993	−0.70	383.1428[M-glc+Na]^+^, 545.1997[M+Na]^+^
4	5-methoxylariciresinol 4-O-*β*-D-glucopyranoside	C_27_H_36_O_12_	15.04	0.89 ± 0.30	575.2112	575.2099	−2.36	412.1434[M-C_10_H_11_O_2_+Na]^+^, 250.0381[M-C_10_H_11_O_2_-glc+Na]^+^, 575.2112[M+Na]^+^
5	2-decenoic acid	C_10_H_18_O_2_	23.41	5.62 ± 1.74	171.1378	171.1380	0.92	55.9342[M-HCOOH-C_5_H_10_+H]^+^, 171.1378[M+H]^+^
6	oploxyne B	C_18_H_30_O_4_	36.91	2.73 ± 0.36	311.2212	311.2217	1.57	107.0527[M-H_2_O-C_11_H_22_O_2_+H]^+^, 79.0547[M-H_2_O-C_11_H_22_O_2_-C_2_H_4_+H]^+^, 311.2212[M+H]^+^
7	oploxyne A	C_17_H_26_O_3_	37.62	2.62 ± 0.44	279.1957	279.1955	−0.82	107.0485 [M-H_2_O-C_10_H_18_O+H]^+^, 79.0545[M-H_2_O-C_10_H_18_O-C_2_H_4_+H]^+^, 279.1957[M+H]^+^
8	oplopantriol B	C_18_H_28_O_3_	37.89	1.28 ± 1.07	293.2110	293.2111	0.41	107.0491[M-H_2_O-C_11_H_20_O+H]^+^, 79.0538[M-H_2_O-C_11_H_20_O-C_2_H_4_+H]^+^, 293.2110[M+H]^+^
9	9,17-octadecadiene-12,14-diyne-1,11,16-triol,1-acetate	C_20_H_28_O_4_	38.63	1.57 ± 1.21	333.2048	333.2060	3.72	105.0700[M-H_2_O-C_13_H_22_O_2_+H]^+^, 79.0546[M-H_2_O-C_13_H_22_O_2_-C_2_H_2_+H]^+^, 333.2048 [M+H]^+^
10	oplopandiol acetate	C_20_H_30_O_4_	39.18	3.93 ± 1.41	335.2215	335.2217	0.56	107.0503[M-H_2_O-C_13_H_22_O_2_+H]^+^, 79.0545[M-H_2_O-C_13_H_22_O_2_-C_2_H_4_+H]^+^, 335.2215[M+H]^+^
11	6,9-octadedicenoic acid	C_18_H_32_O_2_	39.46	5.13 ± 1.95	281.2470	281.2475	1.81	65.0396[M-CH_3_COOH-C_11_H_20_+H]^+^, 281.2470[M+H]^+^
12	falcarindiol	C_17_H_24_O_2_	40.76	101.47 ± 12.16	261.1848	261.1849	0.41	105.0713[M-H_2_O-C_10_H_18_+H]^+^, 79.0548[M-H_2_O-C_10_H_18_-C_2_H_2_+H]^+^, 261.1848 [M+H]^+^
13	oplopandiol	C_17_H_26_O_2_	41.23	119.24 ± 9.28	263.2010	263.2006	−1.69	107.0459[M-H_2_O-C_10_H_18_+H]^+^, 79.0563[M-H_2_O-C_10_H_18_-C_2_H_4_+H]^+^, 263.2010[M+H]^+^
14	falcarinol	C_17_H_24_O	42.85	62.85 ± 7.26	245.1899	245.1900	0.38	105.0699[M-C_10_H_18_+H]^+^, 79.0556[M-C_10_H_18_-C_2_H_2_+H]^+^, 245.1899 [M+H]^+^
15	oplopantriol A	C_18_H_26_O_3_	43.31	67.83 ± 4.40	291.1956	291.1955	0.44	105.0659[M-H_2_O-C_11_H_20_O+H]^+^, 79.0567[M-H_2_O-C_11_H_20_O-C_2_H_2_+H]^+^, 291.1956 [M+H]^+^
16	curcumene	C_15_H_22_	44.92	4.37 ± 2.22	203.1797	203.1794	−1.35	134.1063[M-C_5_H_9_+H]^+^, 65.0382[M-2×C_5_H_9_+H]^+^, 203.1797[M+H]^+^
17	muurolene	C_15_H_24_	45.27	8.27 ± 3.82	205.1950	205.1951	0.38	65.0550[M-2×C_5_H_9_+H]^+^, 205.1950[M+H]^+^
18	oleanolic acid	C_30_H_48_O_3_	46.88	10.83 ± 3.28	479.3508	479.3496	−2.70	231.1715[M-C_16_H_24_O_2_+Na]^+^, 479.3508[M+Na]^+^

Polyynes have been found as the main constituents in the root of *O. elatus* (Yang et al., 2014). Among them, falcarindiol and oplopandiol were determined to have very high contents in the air-dried root bark. As shown in Table 1, polyynes exhibit the same elemental composition and similar MS/MS behaviors, with the characteristic fragment ions at *m/z* 79.05 in the positive ion mode.

For example, the typical protonated molecular ion [M+H]^+^ of FAD was observed at *m/z* 261.1848 in the mass spectrum. The fragment ion at *m/z* 105.0713 was formed by the losses of H_2_O and C_10_H_18_, with *m/z* 79.0548 by further loss of C_2_H_2_. OPD was identified by the protonated molecular ion [M+H]^+^ at *m/z* 263.2010 compared with calculated *m/z* 263.2006. The fragment ion at *m/z* 107.0459 was produced by the losses of H_2_O and C_10_H_18_, and *m/z* 79.0563 was formed by further loss of C_2_H_4_.

In addition, phenylpropanoid compound 4-(3-hydroxyprop-1-en-1-yl)-2,6-dimethoxyphenyl β-D-glucopyranoside was determined to be C_17_H_24_O_9_ at *m/z* 373.1495 ([M+H]^+^, C_17_H_24_O_9_^+^; calculated as 373.1493). The neutral loss of 1 × Glc moiety formed fragment ion at *m/z* 211.1526. Similarly, three lignans were determined by the neutral loss of 1 × Glc moiety in the [M+Na]^+^ mode.

### Detection and identification of metabolites of *O. elatus* extract

The control sample was prepared in parallel, which used in the dilution medium and human fecal microflora, as shown in Fig. 2B. The biotransformed *O. elatus* sample by intestinal bacteria is shown in Fig. 2C. Samples were incubated, pretreated, and analyzed under the same conditions as mentioned in “Incubation of sample in intestinal bacteria”. The potential metabolites were detected from the TIC of the transformed *O. elatus* sample compared to the control group. All the metabolites were further confirmed by the extracted ion chromatograms (EICs) and their MS/MS corresponding fragments. A total of 62 metabolites were identified by UPLC-Q-TOF-MS in the positive mode. Table 2 shows the retention time, signal intensity, experimental and calculated mass *m/z*, difference between *m/z* and calculated *m/z* in ppm, and fragment ions in the MS/MS stage of these 62 metabolites (M1-M62). All these metabolites could not be observed or only in trace amounts in control samples (Schymanski et al., 2014).

**Table 2:** UPLC-Q-TOF/MS data of metabolites detected from the biotransformed *O. elatus* sample in the positive ion mode.

No.	Description	Formula	t_R_ (min)	Signal intensity (×10^5^)	[M+H]^+^ or [M+Na]^+^			Fragment ions in the positive mode with the energy 50 V CID
					*m/z*	Calc *m/z*	Diff (ppm)	
M1	deglycosylation product of 4-(3-hydroxyprop-1-en-1-yl)-2,6-dimethoxyphenyl *β*-D-glucopyranoside	C_11_H_14_O_4_	9.90	2.83 ± 0.32	211.0962	211.0965	1.36	92.0582[M-C_3_H_5_O-OCH_3_×2+H]^+^, 211.0962[M+H]^+^
M2	acetylization product of 9,17-octadecadiene-12,14-diyne-1,11,16-triol,1-acetate	C_22_H_30_O_5_	16.55	16.27 ± 2.46	375.2170	375.2166	−1.07	79.0544[M-C_13_H_24_O_3_-C_2_H_2_+H]^+^, 375.2170[M+H]^+^
M3	deglycosylation product of 4’,7-epoxy-4,9,9’-trihydroxy-3,3’-dimethoxy-5’,8-lignan-4,9-bis[*O*-*β*-D-glucopyranoside]	C_20_H_24_O_6_	20.06	11.37 ± 2.01	383.1470	383.1465	−1.36	188.1603 [M-C_10_H_11_O_4_+Na]^+^, 383.1470 [M+Na]^+^
M4	deglycosylation product of isolariciresinol 3-*O*-*β*-D-glucopyranoside	C_20_H_24_O_6_	20.08	11.32 ± 0.98	383.1464	383.1465	0.30	167.4648 [M-C_10_H_12_O_3_-2×H_2_O+Na]^+^, 383.1464[M+Na]^+^
M5	deglycosylation product of 5-methoxylariciresinol 4-O-β-D-glucopyranoside	C_21_H_26_O_7_	22.65	14.28 ± 1.42	413.1578	413.1571	−1.86	231.8773 [M-C_9_H_10_O_4_+Na]^+^, 413.1578 [M+Na]^+^
M6	hydroxylation product of oplopandiol acetate	C_20_H_30_O_5_	30.58	0.72 ± 0.43	351.2153	351.2166	3.71	107.0840 [M-H_2_O-C_13_H_22_O_3_+H]^+^, 79.0547[M-H_2_O-C_13_H_22_O_3_-C_2_H_4_+H]^+^, 351.2153[M+H]^+^
M7	hydroxylation product of oploxyne B	C_18_H_30_O_5_	30.64	3.02 ± 0.55	327.2163	327.2166	0.92	107.0851 [M-H_2_O-C_11_H_22_O_3_+H]^+^, 79.0543 [M-H_2_O-C_11_H_22_O_3_ -C_2_H_4_+H]^+^, 327.2163[M+H]^+^
M8	acetylization product of oploxyne B	C_20_H_32_O_5_	31.64	1.82 ± 0.63	353.2321	353.2323	0.43	107.0847 [M-H_2_O-C_13_H_24_O_3_+H]^+^, 79.0545[M-H_2_O-C_13_H_24_O_3_-C_2_H_4_+H]^+^, 353.2321[M+H]^+^
M9	hydroxylation product of curcumene	C_15_H_22_O	32.61	42.47 ± 4.29	219.1745	219.1743	0.73	63.0237[M-2×C_5_H_9_-H_2_O+H]^+^, 219.1745 [M+H]^+^
M10	hydroxylation product of 9,17-octadecadiene-12,14-diyne-1,11,16-triol,1-acetate	C_20_H_28_O_5_	34.08	0.98 ± 0.26	349.1994	349.2010	4.45	105.0700[M-H_2_O-C_13_H_22_O_3_+H]^+^, 79.0573[M-H_2_O-C_13_H_22_O_3_-C_2_H_2_+H]^+^, 349.1994 [M+H]^+^
M11	demethylation product of oploxyne B	C_17_H_28_O_4_	34.24	15.83 ± 3.11	297.2061	297.2060	−0.22	107.0508 [M-H_2_O-C_10_H_20_O_2_+H]^+^, 79.0554[M-H_2_O-C_10_H_20_O_2_-C_2_H_4_+H]^+^, 297.2061[M+H]^+^
M12	dehydroxylation product of 2-decenoic acid	C_10_H_18_O	34.27	1.36±0.42	155.1431	155.1430	−0.38	56.9427[M-CHO-C_5_H_10_+H]^+^, 155.1431[M+H]^+^
M13	hydroxylation product of oplopantriol A	C_18_H_26_O_4_	34.90	1.54 ± 0.72	307.1902	307.1904	0.61	105.0682[M-H_2_O-C_11_H_20_O_2_+H]^+^, 79.0539 [M-H_2_O-C_11_H_20_O_2_-C_2_H_2_+H]^+^, 307.1902[M+H]^+^
M14	dehydroxylation product of oplopantriol A	C_18_ H_26_O_2_	34.92	52.73 ± 3.40	275.2003	275.2006	0.94	105.0698[M-H_2_O-C_11_H_20_+H]^+^, 79.0541[M-H_2_O-C_11_H_20_-C_2_H_2_+H]^+^, 275.2003[M+H]^+^
M15	methylation product of falcarindiol	C_18_H_26_O_2_	34.98	49.23 ± 2.49	275.2008	275.2006	−0.89	105.0704[M-H_2_O-C_11_H_20_+H]^+^,79.0548[M-H_2_O-C_11_H_20_-C_2_H_2_+H]^+^, 275.2008[M+H]^+^
M16	dehydroxylation product of oplopandiol acetate	C_20_H_30_O_3_	35.25	37.62 ± 4.21	319.2256	319.2268	3.68	107.0525 [M-H_2_O-C_13_H_22_O+H]^+^, 79.0538[M-H_2_O-C_13_H_22_O-C_2_H_4_+H]^+^, 319.2256[M+H]^+^
M17	hydrogenation product of falcarindiol	C_17_H_26_O_2_	35.89	1.36 ± 0.77	263.2008	263.2006	−0.93	105.0695[M-H_2_O-C_10_H_20_+H]^+^,79.0535[M-H_2_O-C_10_H_20_-C_2_H_2_+H]^+^, 263.2008[M+H]^+^
M18	acetylization product of oplopantriol B	C_20_H_30_O_4_	37.06	12.48 ± 2.41	335.2202	335.2217	4.45	107.0855 [M-H_2_O-C_13_H_22_O_2_+H]^+^,79.0543[M-H_2_O-C_13_H_22_O_2_-C_2_H_4_+H]^+^,335.2202[M+H]^+^
M19	hydrogenation product of oploxyne A	C_18_H_32_O_4_	37.63	7.37 ± 2.49	313.2375	313.2373	−0.53	107.0575[M-H_2_O-C_11_H_24_O_2_+H]^+^, 79.0541[M-H_2_O-C_11_H_24_O_2_-C_2_H_4_+H]^+^, 313.2375[M+H]^+^
M20	hydroxylation product of muurolene	C_15_H_24_O	37.76	13.47 ± 3.57	221.1901	221.1900	−0.49	65.0374[M-2×C_5_H_9_-H_2_O+H]^+^, 221.1901[M+H]^+^
M21	hydrogenation product of 9,17-octadecadiene-12,14-diyne-1,11,16-triol,1-acetate	C_20_H_30_O_4_	37.77	17.38 ± 4.76	335.2204	335.2217	3.85	105.0694[M-H_2_O-C_13_H_24_O_2_+H]^+^, 79.0543[M-H_2_O-C_13_H_24_O_2_-C_2_H_2_+H]^+^, 335.2204 [M+H]^+^
M22	acetylization product of oplopantriol A	C_20_H_28_O_4_	37.90	2.55 ± 0.77	333.2047	333.2060	4.02	105.0705[M-H_2_O-C_13_H_22_O_2_+H]^+^, 79.0524[M-H_2_O-C_13_H_22_O_2_-C_2_H_2_+H]^+^, 333.2047[M+H]^+^
M23	hydrogenation product of oplopandiol acetate	C_20_H_32_O_4_	37.91	12.42 ± 3.28	337.2358	337.2373	4.57	79.0538[M-C_13_H_26_O_3_-C_2_H_4_+H]^+^, 337.2358[M+H]^+^
M24	hydroxylation product of 6,9-octadedicenoic acid	C_18_H_32_O_3_	37.92	17.52 ± 2.71	297.2423	297.2424	0.41	77.0379[M-CH_3_COOH-C_10_H_20_O+H]^+^, 297.2423[M+H]^+^
M25	hydrogenation product of oplopantriol B	C_18_ H_30_O_3_	38.22	24.52 ± 3.98	295.2263	295.2268	1.60	79.0557[M-C_11_H_24_O_2_-C_2_H_4_+H]^+^, 295.2263[M+H]^+^
M26	demethylation product of falcarindiol	C_16_H_22_O_2_	38.59	2.26 ± 0.74	247.1689	247.1693	1.45	105.0686[M-H_2_O-C_9_H_16_+H]^+^, 79.0528[M-H_2_O-C_9_H_16_-C_2_H_2_+H]^+^, 247.1689[M+H]^+^
M27	hydroxylation product of oplopandiol	C_17_H_26_O_3_	38.64	50.12 ± 4.62	279.1958	279.1955	−1.18	107.0496[M-H_2_O-C_10_H_18_O+H]^+^, 79.0541[M-H_2_O-C_10_H_18_O-C_2_H_4_+H]^+^, 279.1958[M+H]^+^
M28	acetylization product of oplopandiol acetate	C_22_H_32_O_5_	39.27	4.92 ± 0.44	377.2326	377.2323	−0.93	79.0543[M-C_15_H_26_O_4_-C_2_H_4_+H]^+^, 377.2326[M+H]^+^
M29	hydroxylation product of falcarinol	C_17_H_24_O_2_	39.54	4.39 ± 0.58	261.1851	261.1849	−0.74	79.0538[M-C_10_H_20_O-C_2_H_2_+H]^+^, 261.1851 [M+H]^+^
M30	hydroxylation product of oploxyne A	C_17_H_26_O_4_	39.60	2.74 ± 0.94	295.1902	295.1904	0.63	79.0557[M-C_10_H_20_O_3_-C_2_H_4_+H]^+^, 295.1902[M+H]^+^
M31	demethylation product of falcarinol	C_16_H_22_O	39.61	1.02 ± 0.57	231.1744	231.1743	−0.25	79.0544[M-C_9_H_18_-C_2_H_2_+H]^+^, 231.1744[M+H]^+^
M32	demethylation product of oplopantriol B	C_17_H_26_O_3_	39.69	33.79 ± 4.95	279.1965	279.1955	−3.70	79.0542[M-C_10_H_20_O_2_-C_2_H_4_+H]^+^, 279.1965[M+H]^+^
M33	hydrogenation product of oploxyne A	C_17_H_28_O_3_	40.06	10.32 ± 1.45	281.2113	281.2111	−0.64	79.0541[M-C_10_H_22_O_2_-C_2_H_4_+H]^+^, 281.2113[M+H]^+^
M34	hydroxylation product of oplopantriol B	C_18_H_28_O_4_	40.07	5.28 ± 1.82	309.2062	309.2060	−0.53	79.0560[M-C_11_H_22_O_3_-C_2_H_4_+H]^+^, 309.2062[M+H]^+^
M35	demethoxy product of oploxyne B	C_17_H_28_O_3_	40.07	9.24 ± 1.23	281.2112	281.2111	−0.28	79.0541[M -C_10_H_22_O_2_-C_2_H_4_+H]^+^, 281.2112[M+H]^+^
M36	hydrogenation product of oplopantriol A	C_18_ H_28_O_3_	40.09	2.83 ± 0.75	293.2109	293.2111	0.76	79.0538 [M-C_11_H_24_O_2_-C_2_H_2_+H]^+^, 293.2109[M+H]^+^
M37	demethylation product of oleanolic acid	C_29_H_46_O_3_	40.18	0.54 ± 0.26	443.3524	443.3520	−0.97	165.0912[M-C_16_H_24_O_2_-2×CH_3_+H]^+^, 443.3524[M+H]^+^
M38	demethylation product of curcumene	C_14_H_20_	40.18	2.42 ± 0.29	189.1636	189.1638	0.94	51.0229[M-2×C_5_H_9_+H]^+^, 189.1636[M+H]^+^
M39	dehydroxylation product of oploxyne A	C_17_H_26_O_2_	40.29	0.51 ± 0.23	263.2005	263.2006	0.22	79.0552[M-C_10_H_20_O-C_2_H_4_+H]^+^, 263.2005[M+H]^+^
M40	demethylation product of oplopandiol	C_16_H_24_O_2_	40.48	7.12 ± 0.44	249.1851	249.1849	−0.78	107.0863[M-H_2_O-C_9_H_14_+H]^+^, 79.0543[M-H_2_O-C_9_H_14_-C_2_H_4_+H]^+^, 249.1851[M+H]
M41	hydrogenation product of falcarinol	C_17_H_26_O	40.50	22.15 ± 2.35	247.2053	247.2056	1.39	79.0528[M-C_10_H_22_-C_2_H_2_+H]^+^, 247.2053 [M+H]^+^
M42	methylation product of oplopandiol acetate	C_21_H_32_O_4_	40.51	1.10 ± 0.77	349.2373	349.2373	0.10	79.0541[M-C_14_H_26_O_3_-C_2_H_4_+H]^+^, 349.2373[M+H]^+^
M43	dehydroxylation product of oplopandiol	C_17_H_26_O	40.53	20.63 ± 3.86	247.2060	247.2056	−1.45	107.0513[M-C_10_H_20_+H]^+^, 79.0528[M-C_10_H_20_-C_2_H_4_+H]^+^, 247.2060[M+H]^+^
M44	dehydroxylation product of falcarinol	C_17_H_24_	40.53	4.76 ± 1.42	229.1953	229.1951	−0.98	77.0398[M-C_9_H_18_-C_2_H_2_+H]^+^, 229.1953 [M+H]^+^
M45	demethylation product of muurolene	C_14_H_22_	40.55	4.97 ± 0.99	191.1796	197.1794	−0.91	53.0384[M-2×C_5_H_9_+H]^+^, 191.1796 [M+H]^+^
M46	dehydroxylation product of falcarindiol	C_17_H_24_O	41.18	67.22 ± 3.74	245.1903	245.1900	−1.26	105.0692[M-C_10_H_20_+H]^+^, 79.0521[M-C_10_H_20_-C_2_H_2_+H]^+^, 245.1903[M+H]^+^
M47	methylation product of oplopantriol A	C_19_H_28_O_3_	41.41	0.52 ± 0.28	305.2111	305.2113	−0.59	79.0547[M-C_12_H_24_O_2_-C_2_H_2_+H]^+^, 305.2111 [M+H]^+^
M48	demethylation product of oplopantriol A	C_17_H_24_O_3_	41.42	53.27 ± 3.42	277.1800	277.1798	−0.65	79.0537[M-C_10_H_20_O_2_-C_2_H_2_+H]^+^, 277.1800[M+H]^+^
M49	methylation product of oplopandiol	C_18_H_28_O_2_	41.43	51.35 ± 2.53	277.2160	277.2162	0.75	107.0871[M-H_2_O-C_11_H_20_+H]^+^, 79.0547[M-H_2_O-C_11_H_20_-C_2_H_4_+H]^+^, 277.2160[M+H]^+^
M50	dehydroxylation product of oplopantriol B	C_18_H_28_O_2_	41.44	53.87 ± 4.21	277.2164	277.2162	−0.70	79.0541[M-C_11_H_22_O-C_2_H_4_+H]^+^, 277.2164[M+H]^+^
M51	methylation product of falcarinol	C_18_H_26_O	41.59	3.75 ± 0.89	259.2057	259.2056	−0.23	79.0524[M-C_11_H_22_-C_2_H_2_+H]^+^, 259.2057 [M+H]^+^
M52	methylation product of oplopantriol B	C_19_ H_30_O_3_	41.69	1.26 ± 0.82	307.2262	307.2268	1.87	79.0543[M-C_12_H_24_O_2_-C_2_H_4_+H]^+^, 307.2262[M+H]^+^
M53	methylation product of oploxyne B	C_19_H_32_O_4_	41.76	0.37 ± 0.20	325.2371	325.2373	0.73	79.0540[M-C_12_H_26_O_3_ -C_2_H_4_+H]^+^, 325.2371[M+H]^+^
M54	dehydroxylation product of 9,17-octadecadiene-12,14-diyne-1,11,16-triol,1-acetate	C_20_H_28_O_3_	42.15	28.46 ± 1.55	317.2096	317.2111	4.81	79.0537[M-C_13_H_24_O_2_-C_2_H_2_+H]^+^, 317.2096 [M+H]^+^
M55	dehydroxylation product of oploxyne B	C_18_H_30_O_3_	42.16	56.82 ± 2.47	295.2273	295.2268	−1.80	79.0550 [M-C_11_H_24_O_2_ -C_2_H_4_+H]^+^, 295.2273[M+H]^+^
M56	hydroxylation product of falcarindiol	C_17_H_24_O_3_	42.20	79.26 ± 1.34	277.1797	277.1798	0.44	105.0693[M-H_2_O-C_10_H_18_O+H]^+^,79.0541[M-H_2_O-C_10_H_18_O-C_2_H_2_+H]^+^, 277.1797[M+H]^+^
M57	methylation product of 9,17-octadecadiene-12,14-diyne-1,11,16-triol,1-acetate	C_21_H_30_O_4_	42.52	0.42 ± 0.28	347.2213	347.2217	1.11	79.0541[M-C_14_H_26_O_3_-C_2_H_2_+H]^+^, 347.2213 [M+H]^+^
M58	hydrogenation product of curcumene	C_15_H_24_	42.65	70.36 ± 4.29	205.1953	205.1951	−1.09	67.0540[M-2×C_5_H_9_+H]^+^, 205.1953[M+H]^+^
M59	demethylation product of 6,9-octadedicenoic acid	C_17_H_30_O_2_	42.75	0.48 ± 0.32	267.2327	267.2319	−3.17	65.0390[M-CH_3_COOH-C_10_H_18_+H]^+^, 267.2327[M+H]^+^
M60	dehydroxylation product of 6,9-octadedicenoic acid	C_18_H_32_O	43.40	65.77 ± 5.21	265.2536	265.2526	−3.81	69.0688[M-CH_3_CHO-C_11_H_20_+H]^+^, 265.2536[M+H]^+^
M61	hydrogenation product of 6,9-octadedicenoic acid	C_18_H_34_O_2_	43.50	128.46 ± 8.42	283.2636	283.2632	−1.57	65.0389[M-CH_3_COOH-C_11_H_22_+H]^+^, 283.2636[M+H]^+^
M62	acetylization product of oleanolic acid	C_32_H_50_O_4_	48.23	2.48 ± 1.63	521.3606	521.3601	−0.94	220.0842[M-C_16_H_24_O_2_-C_2_H_3_O+Na]^+^, 521.3606[M+Na]^+^

#### Polyynes

A total of 46 metabolites of nine polyynes generated by the transformation of human intestinal microflora were detected and identified. For each polyyne, at least four types of metabolites were identified. Due to the high biological activities, FAD and OPD selected as the representative compounds of polyynes were stated in detail.

**Figure 3:**
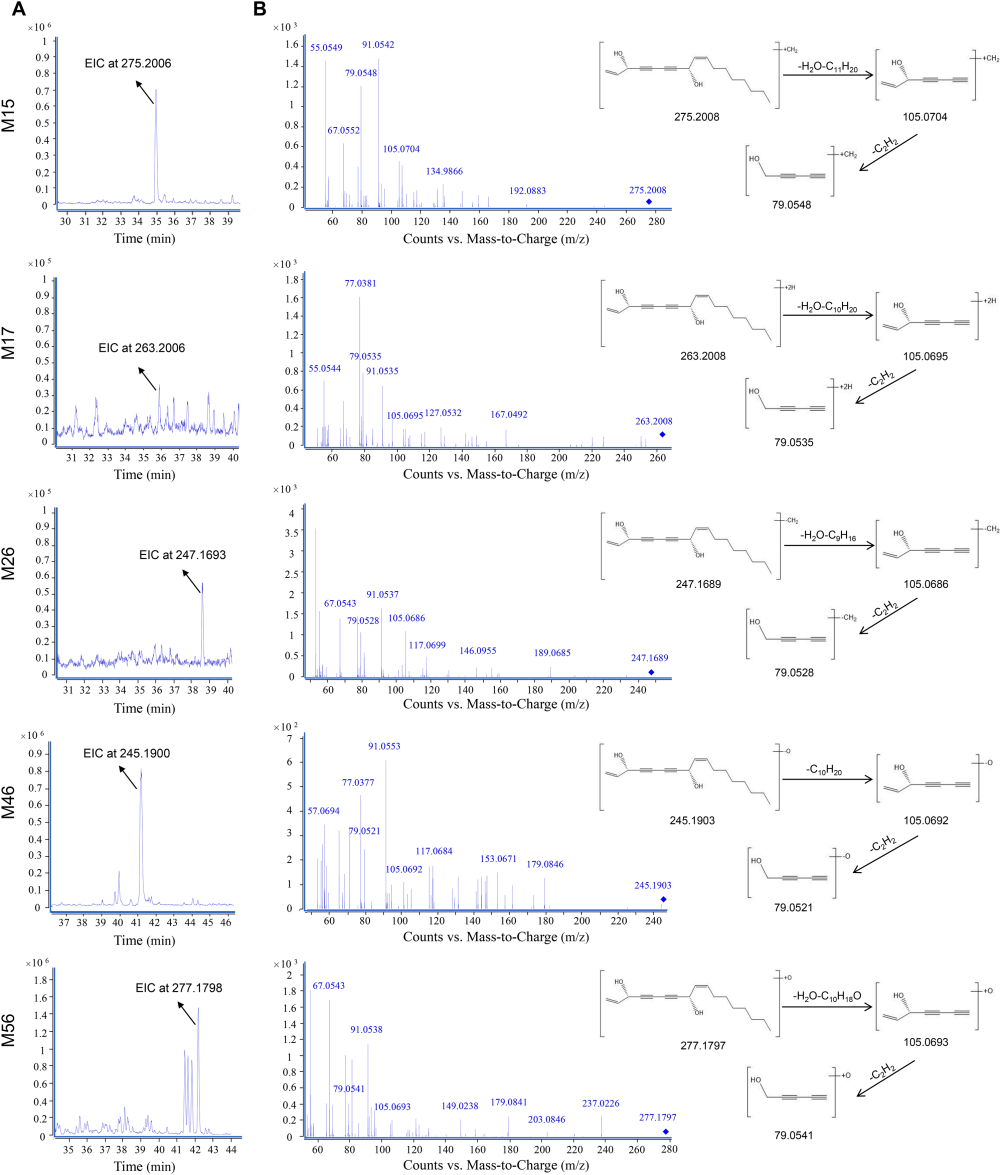
Metabolites of falcarindiol using UPLC-TOF/MS in the positive ion mode. (A) Extracted ion chromatograms (EICs); (B) MS/MS spectra and structural elucidation.

The EICs and MS/MS spectrums of metabolites of FAD are shown in Fig. 3. Five metabolites including M15, M17, M26, M46, M56 were detected. M15 was assigned to be the methylation product of FAD with the molecular formula C_18_H_26_O_2_ at *m/z* 275.2008 ([M+H]^+^, C_18_H_26_O_2_^+^; calculated as 275.2006). The fragment ion at *m/z* 105.0704 was generated by the neutral losses of H_2_O and C_11_H_20_, and *m/z* 79.0548 was formed by further loss of C_2_H_2_. M17 was assigned to be the hydrogenation product of FAD with the characteristic fragment ions at *m/z* 105.0695 and 79.0535. In addition, metabolites M26, M46, and M56 were assigned as demethylation, dehydroxylation, and hydroxylation products of FAD, respectively.

**Figure 4:**
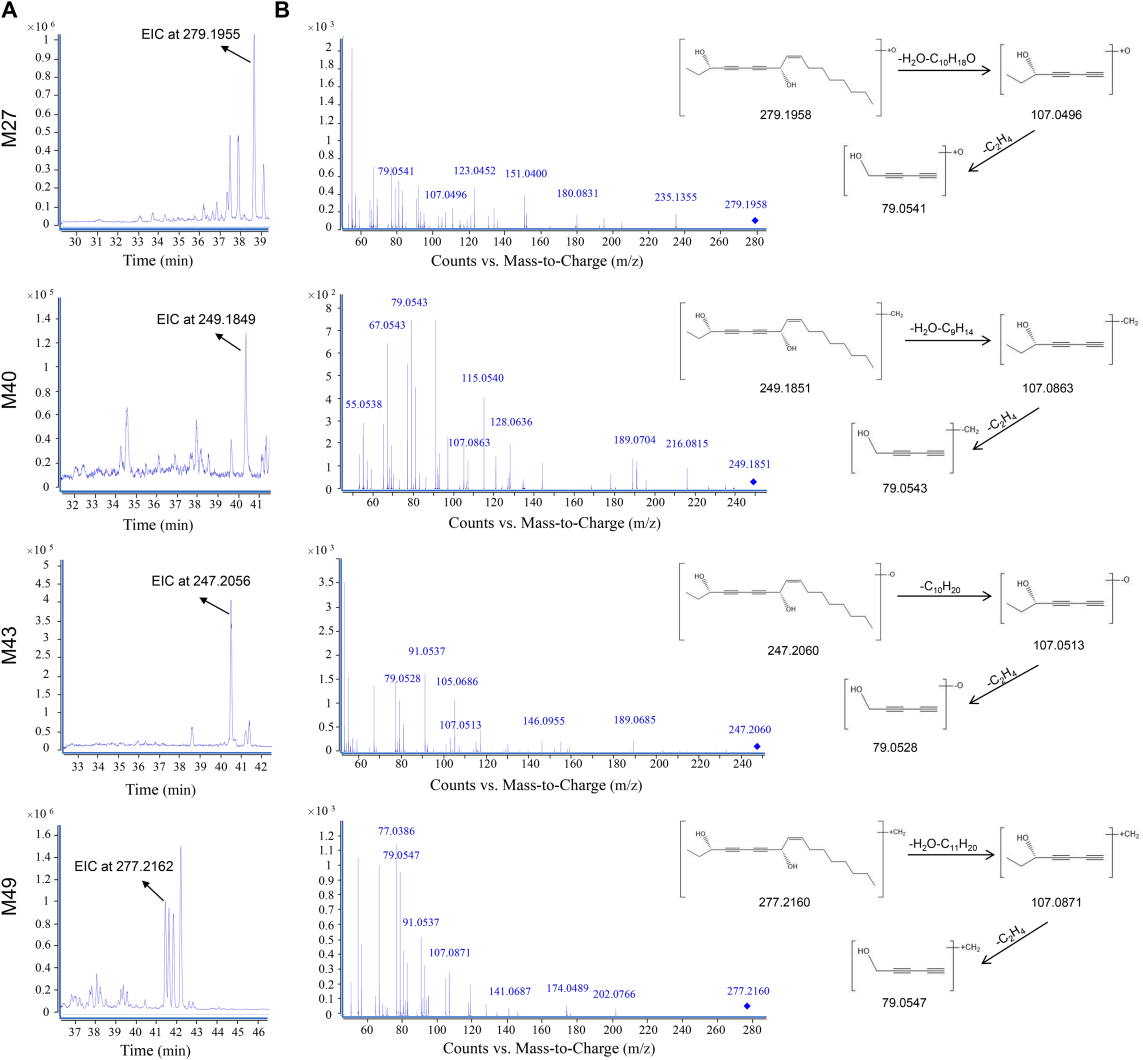
Metabolites of oplopandiol using UPLC-TOF/MS in the positive ion mode. (A) EICs; (B) MS/MS spectra and structural elucidation.

Figure 4 presents the EICs and MS/MS spectrums of OPD metabolites (M27, M40, M43, and M49). M27 was assigned as the hydroxylation product of OPD, owing to the presence of [M+H]^+^ at *m/z* 279.1958. The characteristic fragment ion at *m/z* 107.0496 was formed by the neutral losses of H_2_O and C_10_H_18_O, and *m/z* 79.0541 was formed by further loss of C_2_H_4_. Similarly, three other metabolites like M40, M43, and M49 were supposed to be the demethylation, dehydroxylation, and methylation products of OPD.

#### Lignans

M3–M5 were the deglycosylation products of three lignans *via* the loss of glycose moieties. For example, the parent compound of M3 was determined to be C_32_H_44_O_16_ while M3 was C_20_H_24_O_6_, indicating M3 was the deglycosylation product *via* the loss of two glucose moieties. M4 and M5 were assigned as the products by losing a glucose moiety from their corresponding parent lignan compounds.

#### Phenylpropanoids

M1 was identified as the deglycosylation metabolite of phenylpropanoid compound 4-(3-hydroxyprop-1-en-1-yl)-2,6-dimethoxyphenyl β-D-glucopyranoside. The protonated molecular ion [M+H]^+^ of M1 at *m/z* 211.0962 was observed in the positive ion mode, providing the molecular formula of C_11_H_14_O_4_.

#### Others

For two sesquiterpenes, M9, M38, and M58 were identified as the hydroxylation, demethylation, and hydrogenation products of curcumene, while M20 and M45 were the hydroxylation and demethylation product of muurolene, respectively. M37 and M62 were identified as the demethylation and acetylization products of oleanolic acid. In addition, for 2 fatty acids, M12 was the dehydroxylation product of 2-decenoic acid, while M24 and M59-61 were the products of 6,9-octadedicenoic acid.

### Proposed metabolic pathways of *O. elatus* extract

**Figure 5:**
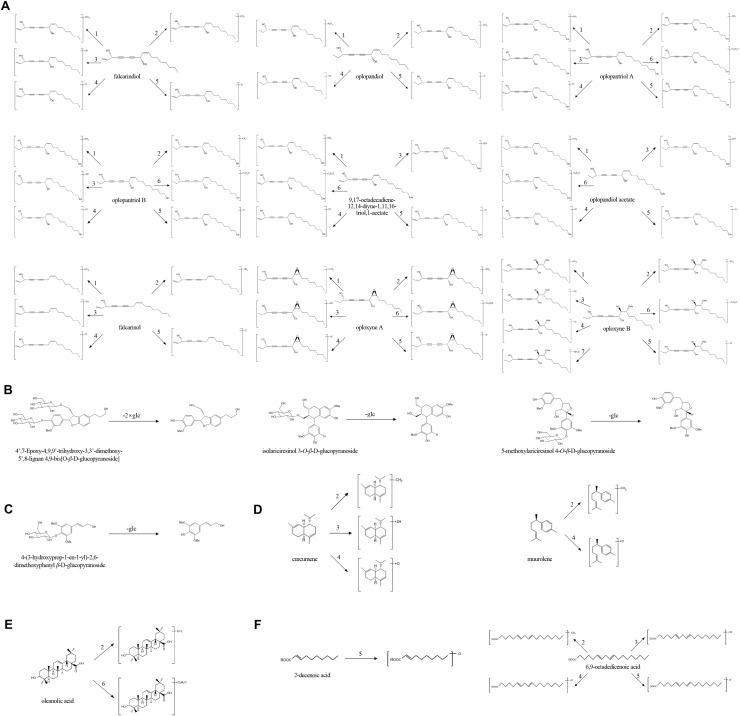
The proposed metabolic pathways of *O. elatus* extract by human intestinal microflora, including (A) Polyynes; (B) Lignans; (C) Phenylpropanoid; (D) Sesquiterpenes; (E) Triterpenoid; (F) Fatty acids. Methylation (1), demethylation (2), hydrogenation (3), hydroxylation (4), dehydroxylation (5), acetylization (6) and demethoxylation (7) were observed in this biotransformation.

The proposed metabolic pathways of *O. elatus* extract by human intestinal microflora are presented in Fig. 5. Multiple major metabolite pathways can be observed in this study. The common pathways involved in the biotransformation of *O. elatus* extract include methylation, demethylation, hydroxylation, dehydroxylation, acetylation, hydrogenation, demethoxylation, and deglycosylation. Among them, polyynes were undoubtedly the most important compounds, as 46 out of 62 metabolites originated from polyynes. By comparing the signal intensity of metabolites, we could find that methylation, dehydroxylation and hydroxylation are major metabolic pathways of polyynes. Moreover, four metabolites of lignans and phenylpropanoid were produced by the loss of glucose. The other metabolites were generated from one triterpenoid and two fatty acids. This indicated that polyynes of *O. elatus* generated comprehensive biotransformation and were more readily metabolized than other compounds under the same conditions.

In summary, the main metabolic pathways of *O. elatus* refer to hydrolytic and reductive reactions by gut microorganisms. Because of the complexity of active ingredients or constituent concentrations, *in vivo* exposure, and individual differences, the metabolic profiles of *O. elatus* might be affected by several factors.

## Discussion

In this study, a UPLC-Q-TOF-MS/MS method was developed to screen and identify the chemical composition and metabolites from a traditional Chinese herb, the air-dried root bark of *O. elatus*. A total of 18 ingredients and 62 metabolites biotransformed by human intestinal microflora were characterized from *O. elatus* in UPLC-Q-TOF/MS positive ion mode. Two polyynes, falcarindiol and oplopandiol, as the main components of *O. elatus* and their metabolites by human intestinal microflora are mainly illustrated. It could be noted that the major metabolic pathways of *O. elatus* refer to methylation, dehydroxylation, and hydroxylation. Studies on the chemical and metabolic profiling of *O. elatus* by human intestinal microflora will be helpful for the understanding of mechanism research on the active components and further *in vivo* investigation.

## Supplemental Information

10.7717/peerj.12513/supp-1 Supplemental Information 1 Raw data. The mass spectrum of *Oplopanax elatus* (the herbal medication we studied) and its metabolites by human intestinal microbiota, which were detected by an Agilent 6545 Q-TOF-MS system. Mass data including MS and MS/MS information could be analyzed by Agilent MassHunter Workstation software. The software was applied in the screening and identification of the probable compounds, based on the accurate measurements of m/z values with databases.

## Additional Information and Declarations

### Data Availability

The following information was supplied regarding data availability: The raw measurements are available in the Supplementary Files.

## References

[ref-1] Barko PC, McMichael MA, Swanson KS, Williams DA (2018). The gastrointestinal microbiome: a review. Journal of Veterinary Internal Medicine.

[ref-2] Bäckhed F, Ding H, Wang T, Hooper LV, Koh GY, Nagy A, Semenkovich CF, Gordon JI (2004). The gut microbiota as an environmental factor that regulates fat storage. Proceedings of the National Academy of Sciences of the United States of America.

[ref-3] Chekmeneva E, Dos Santos Correia G, Gómez-Romero M, Stamler J, Chan Q, Elliott P, Nicholson JK, Holmes E (2018). Ultra-performance liquid chromatography-high-resolution mass spectrometry and direct infusion-high-resolution mass spectrometry for combined exploratory and targeted metabolic profiling of human urine. Journal of Proteome Research.

[ref-4] Chen F, Wen Q, Jiang J, Li HL, Tan YF, Li YH, Zeng NK (2016). Could the gut microbiota reconcile the oral bioavailability conundrum of traditional herbs?. Journal of Ethnopharmacology.

[ref-5] Dai SX, Li WX, Han FF, Guo YC, Zheng JJ, Liu JQ, Wang Q, Gao YD, Li GH, Huang JF (2016). In silico identification of anti-cancer compounds and plants from traditional Chinese medicine database. Scientific Reports.

[ref-6] Defois C, Ratel J, Garrait G, Denis S, Le Goff O, Talvas J, Mosoni P, Engel E, Peyret P (2018). Food chemicals disrupt human gut microbiota activity and impact intestinal homeostasis as revealed by in vitro systems. Scientific Reports.

[ref-7] Dou DQ, Hu XY, Zhao YR, Kang TG, Liu FY, Kuang HX, Smith DC (2009). Studies on the anti-psoriasis constituents of Oplopanax elatus Nakai. Natural Product Research.

[ref-8] Du LY, Tao JH, Jiang S, Qian DW, Guo JM, Duan JA (2017). Metabolic profiles of the Flos Abelmoschus manihot extract by intestinal bacteria from the normal and CKD model rats based on UPLC-Q-TOF/MS. Biomedical Chromatography.

[ref-9] Eom S, Lee J, Kim H, Hyun T (2017). De novo transcriptomic analysis to reveal functional genes involved in triterpenoid saponin biosynthesis in Oplopanax elatus NAKAI. Journal of Applied Botany and Food Quality.

[ref-10] Gao MX, Tang XY, Zhang FX, Yao ZH, Yao XS, Dai Y (2018). Biotransformation and metabolic profile of Xian-Ling-Gu-Bao capsule, a traditional Chinese medicine prescription, with rat intestinal microflora by ultra-performance liquid chromatography coupled with quadrupole time-of-flight tandem mass spectrometry analysis. Biomedical Chromatography.

[ref-11] Huang WH, Shao L, Wang CZ, Yuan CS, Zhou HH (2014a). Anticancer activities of polyynes from the root bark of Oplopanax horridus and their acetylated derivatives. Molecules.

[ref-12] Huang W, Yang J, Zhao J, Wang CZ, Yuan CS, Li SP (2010). Quantitative analysis of six polyynes and one polyene in Oplopanax horridus and Oplopanax elatus by pressurized liquid extraction and on-line SPE-HPLC. Journal of Pharmaceutical and Biomedical Analysis.

[ref-13] Huang WH, Zhang QW, Yuan CS, Wang CZ, Li SP, Zhou HH (2014b). Chemical constituents of the plants from the genus Oplopanax. Chemistry & Biodiversity.

[ref-14] Jin MM, Zhang WD, Jiang HH, Du YF, Guo W, Cao L, Xu HJ (2018). UPLC-Q-TOF-MS/MS-guided dereplication of Pulsatilla chinensis to identify triterpenoid saponins. Phytochemical Analysis.

[ref-15] Knispel N, Ostrozhenkova E, Schramek N, Huber C, Peña-Rodríguez LM, Bonfill M, Palazón J, Wischmann G, Cusidó RM, Eisenreich W (2013). Biosynthesis of panaxynol and panaxydol in Panax ginseng. Molecules.

[ref-16] Koppel N, Maini Rekdal V, Balskus EP (2017). Chemical transformation of xenobiotics by the human gut microbiota. Science.

[ref-17] Li W, Hong B, Li Q, Li Z, Bi K (2019). An integrated serum and urinary metabonomic research of Rhizoma Curcumae-Rhizoma Sparganii drug pair in hysteromyoma rats based on UPLC-Q-TOF-MS analysis. Journal of Ethnopharmacology.

[ref-18] Lou Y, Zheng J, Hu H, Lee J, Zeng S (2015). Application of ultra-performance liquid chromatography coupled with quadrupole time-of-flight mass spectrometry to identify curcumin metabolites produced by human intestinal bacteria. Journal of Chromatography B Analytical Technologies in the Biomedical and Life Sciences.

[ref-19] Moon HK, Kim YW, Hong YP, Park SY (2013). Improvement of somatic embryogenesis and plantlet conversion in Oplopanax elatus, an endangered medicinal woody plant. Springerplus.

[ref-20] Pagliari D, Gambassi G, Piccirillo CA, Cianci R (2017). The intricate link among gut “immunological niche,” microbiota, and xenobiotics in intestinal pathology. Mediators of Inflammation.

[ref-21] Panossian AG, Efferth T, Shikov AN, Pozharitskaya ON, Kuchta K, Mukherjee PK, Banerjee S, Heinrich M, Wu W, Guo DA, Wagner H (2021). Evolution of the adaptogenic concept from traditional use to medical systems: pharmacology of stress- and aging-related diseases. Medicinal Research Reviews.

[ref-22] Purup S, Larsen E, Christensen LP (2009). Differential effects of falcarinol and related aliphatic C(17)-polyacetylenes on intestinal cell proliferation. Journal of Agricultural and Food Chemistry.

[ref-23] Qiao X, Sun W, Wang C, Zhang L, Li P, Wen X, Yang J, Yuan C (2017). Polyyne-enriched extract from oplopanax elatus significantly ameliorates the progression of colon carcinogenesis in Apc(Min/+) mice. Molecules.

[ref-24] Rajilić-Stojanović M, de Vos WM (2014). The first 1,000 cultured species of the human gastrointestinal microbiota. FEMS Microbiology Reviews.

[ref-25] Schymanski EL, Jeon J, Gulde R, Fenner K, Ruff M, Singer HP, Hollender J (2014). Identifying small molecules via high resolution mass spectrometry: communicating confidence. Environmental Science & Technology.

[ref-26] Shao L, Nie MK, Chen MY, Wang J, Wang CZ, Huang WH, Yuan CS, Zhou HH (2016). Screening and identifying antioxidants from Oplopanax elatus using 2,2’-diphenyl-1-picrylhydrazyl with off-line two-dimensional HPLC coupled with diode array detection and tandem time-of-flight mass spectrometry. Journal of Separation Science.

[ref-27] Shikov AN, Pozharitskaya ON, Makarov VG, Yang WZ, Guo DA (2014). Oplopanax elatus (Nakai) Nakai: chemistry, traditional use and pharmacology. Chinese Journal of Natural Medicines.

[ref-28] Sun W, He YS, Xu LH, Zhang BY, Qi LW, Yang J, Li P, Wen XD (2016). Pharmacokinetic profiles of falcarindiol and oplopandiol in rats after oral administration of polyynes extract of Oplopanax elatus. Chinese Journal of Natural Medicines.

[ref-29] Tao JH, Duan JA, Jiang S, Qian YY, Qian DW (2016). Biotransformation and metabolic profile of buddleoside with human intestinal microflora by ultrahigh-performance liquid chromatography coupled to hybrid linear ion trap/orbitrap mass spectrometer. Journal of Chromatography B, Analytical Technologies in the Biomedical and Life Sciences.

[ref-30] Teschke R, Wolff A, Frenzel C, Eickhoff A, Schulze J (2015). Herbal traditional Chinese medicine and its evidence base in gastrointestinal disorders. World Journal of Gastroenterology.

[ref-31] Thursby E, Juge N (2017). Introduction to the human gut microbiota. Biochemical Journal.

[ref-32] Wang X, Sun W, Sun H, Lv H, Wu Z, Wang P, Liu L, Cao H (2008). Analysis of the constituents in the rat plasma after oral administration of Yin Chen Hao Tang by UPLC/Q-TOF-MS/MS. Journal of Pharmaceutical and Biomedical Analysis.

[ref-33] Wang CZ, Wan JY, Wan J, Wang S, Luo Y, Zeng J, Yao H, Zhang CF, Zhang QH, Sawadogo WR, Xu M, Du W, Qi LW, Li P, Yuan CS (2020). Human intestinal microbiota derived metabolism signature from a North American native botanical Oplopanax horridus with UPLC/Q-TOF-MS analysis. Biomedical Chromatography.

[ref-34] Wewer V, Dombrink I, vom Dorp K, Dörmann P (2011). Quantification of sterol lipids in plants by quadrupole time-of-flight mass spectrometry. Journal of Lipid Research.

[ref-35] Wu Y, Wang P, Yang H, Sui F (2019). UPLC-Q-TOF-MS and UPLC-MS/MS methods for metabolism profiles and pharmacokinetics of major compounds in Xuanmai Ganjie Granules. Biomedical Chromatography.

[ref-36] Yang MC, Kwon HC, Kim YJ, Lee KR, Yang HO (2010). Oploxynes A and B, polyacetylenes from the stems of Oplopanax elatus. Journal of Natural Products.

[ref-37] Yang M, Lao L (2019). Emerging applications of metabolomics in traditional chinese medicine treating hypertension: biomarkers, pathways and more. Frontiers in Pharmacology.

[ref-38] Yang S, Tian M, Yuan L, Deng H, Wang L, Li A, Hou Z, Li Y, Zhang Y (2016). Analysis of E. rutaecarpa Alkaloids constituents in vitro and in vivo by UPLC-Q-TOF-MS combined with diagnostic fragment. Journal of Analytical Methods in Chemistry.

[ref-39] Yang ZM, Zhao J, Lao KM, Chen XJ, Leong F, Wang CZ, Yuan CS, Li SP (2014). Determination of six polyynes in Oplopanax horridus and Oplopanax elatus using polyethylene glycol modified reversed migration microemulsion electrokinetic chromatography. Electrophoresis.

